# How to Define and Meet Blood Pressure Targets After Traumatic Brain Injury: A Narrative Review

**DOI:** 10.1007/s12028-024-02048-5

**Published:** 2024-07-09

**Authors:** Ahmet Kartal, Chiara Robba, Adel Helmy, Stefan Wolf, Marcel J. H. Aries

**Affiliations:** 1 University Hospital Heidelberg, Heidelberg University, Heidelberg, Germany; 2 Anesthesia and Intensive Care, IRCCS Policlinico San Martino, Genoa, Italy; 3 Department of Surgical Sciences and Integrated Sciences, University of Genoa, Genoa, Italy; 4 Division of Neurosurgery, Department of Clinical Neurosciences, Addenbrooke’s Hospital, University of Cambridge, Cambridge, UK; 5 Department of Neurosurgery, Charité - Universitätsmedizin Berlin, Berlin, Germany; 6 Department of Intensive Care Medicine, Maastricht University Medical Center, Maastricht University, Maastricht, The Netherlands; 7 Institute of Mental Health and Neurosciences, University Maastricht, Maastricht, The Netherlands

**Keywords:** Cerebral perfusion dynamics, Clinical guidelines, Intracranial pressure, Neuromonitoring, Blood pressure monitoring, Personalized hemodynamic management, Traumatic brain injury

## Abstract

**Background:**

Traumatic brain injury (TBI) poses a significant challenge to healthcare providers, necessitating meticulous management of hemodynamic parameters to optimize patient outcomes. This article delves into the critical task of defining and meeting continuous arterial blood pressure (ABP) and cerebral perfusion pressure (CPP) targets in the context of severe TBI in neurocritical care settings.

**Methods:**

We narratively reviewed existing literature, clinical guidelines, and emerging technologies to propose a comprehensive approach that integrates real-time monitoring, individualized cerebral perfusion target setting, and dynamic interventions.

**Results:**

Our findings emphasize the need for personalized hemodynamic management, considering the heterogeneity of patients with TBI and the evolving nature of their condition. We describe the latest advancements in monitoring technologies, such as autoregulation-guided ABP/CPP treatment, which enable a more nuanced understanding of cerebral perfusion dynamics. By incorporating these tools into a proactive monitoring strategy, clinicians can tailor interventions to optimize ABP/CPP and mitigate secondary brain injury.

**Discussion:**

Challenges in this field include the lack of standardized protocols for interpreting multimodal neuromonitoring data, potential variability in clinical decision-making, understanding the role of cardiac output, and the need for specialized expertise and customized software to have individualized ABP/CPP targets regularly available. The patient outcome benefit of monitoring-guided ABP/CPP target definitions still needs to be proven in patients with TBI.

**Conclusions:**

We recommend that the TBI community take proactive steps to translate the potential benefits of personalized ABP/CPP targets, which have been implemented in certain centers, into a standardized and clinically validated reality through randomized controlled trials.

## Primary and Secondary Brain Injury

Traumatic brain injury (TBI) results from high-energy forces that lead to dysfunction and/or damage to the brain parenchyma. The initial action of forces on the brain results in so-called primary brain injury, causing direct neuronal damage in the early posttrauma period. The epidemiology of severe TBI has changed over recent years. In developed countries, there are fewer road traffic accidents but more admissions after falls, which mostly concern older patients. Older individuals frequently exhibit a higher prevalence of comorbidities, for example, hypertension, and a reduced cardiopulmonary reserve [1]. Medications, such as anticoagulation or antiplatelet therapy, can increase the risk of bleeding and expansion of intracranial hematoma, thus aggravating neuronal damage further. These factors have contributed to the complexity of the current treatment of patients with severe TBI. Experts in the field of TBI stress that treatment should be determined individually, but at the moment, this has not led to clinical studies that warrant changes in the Brain Trauma Foundation guidelines [1, 2].

The primary brain injury at the trauma scene, by definition, requires a preventive approach. However, changes later on—coined “secondary brain injury”—are theoretically amenable to medical and surgical intervention to improve patient outcomes. It is characterized by additional dysfunction and decay of brain cells through the generation of free radicals, prolonged cell depolarization, tissue as well as cell edema, excitotoxicity due to increased neurotransmitter concentrations, disruption of the blood–brain barrier (BBB), disturbed calcium homeostasis, impaired cerebral autoregulation mechanisms, activation of coagulation and inflammatory pathways, mitochondrial dysfunction, and expansion of intracranial hematomas [3, 4]. The complexity of the pathophysiological changes after TBI has become clearer over recent years but has proven difficult to catch or describe with currently available imaging and neuromonitoring techniques (Stocchetti et al. [5]). Unfortunately, the increased pathophysiological insight so far has not led to new prospective studies able to show a reduction in secondary damage and/or improve the outcome of patients with severe TBI [1].

One study participant who regained new interest is the need for the brain’s continuous supply of oxygen and nutrients and the ways this can be supported bedside. Like many other diseases, randomized studies testing the benefits of “low” versus “high” arterial blood pressure (ABP) targets during disease course on patient outcomes have produced disappointing results. These studies do not provide guidance on the lowest tolerable ABP or cerebral perfusion pressure (CPP) for a given patient. This is particularly challenging as it requires considering the potential side effects of vasopressors and fluids. However, recent trial results from perioperative studies employing individualized ABP targets have strengthened the plausible association between cerebral hypoperfusion and unfavorable patient outcomes [6, 7].

In this focused review, we will briefly discuss a common approach for defining “adequate” cerebral perfusion based on arbitrary or population-driven thresholds. We then will discuss novel methods for potentially defining “optimal” ABP or CPP targets individually based on monitoring cerebral blood flow autoregulation or cerebrovascular pressure reactivity. Finally, we put the findings into perspective and discuss future directions.

## Initial Resuscitation

High-energy trauma patients are cared for by a multidisciplinary team according to the “Advanced Trauma Life Support (ATLS)” principle, which tries to identify and treat all life-treating injuries as quickly as possible. Directly after the trauma ictus (scene, transport, or emergency department), it is advised to keep the mean arterial blood pressure (MAP) above 80 mm Hg or to keep the systolic blood pressure above 100 mm Hg for patients with TBI aged 50–69 and above 110 mm Hg for younger (15–49 years) or older (> 70 years old) patients [8]. A recent study studied the relationship between hypotension status and mortality in 12.582 adult study participants with TBI during prehospital and hospital (emergency room) resuscitation. In the group with “no” hypotension, mortality was 9.2% (95%-confidence interval (CI) 8.7–9.8%), with prehospital hypotension “only” 27.8% (95%-CI 24.6–31.2%), with hospital hypotension “only” 45.6% (95%-CI 39.1–52.1%) and “combined” prehospital/hospital hypotension 57.6% (95%-CI 50.0–65.0%, (*p* < 0.0001)). The adjusted odds ratio for mortality reflected the same progression: 1.0 (reference: no hypotension), 1.8 (95%-CI 1.39–2.33), 2.61 (95%-CI 1.73–3.94), and 4.36 (955-CI 2.78–6.84), respectively [9]. Statewide implementation of the prehospital TBI guidelines (avoidance/treatment of hypoxia, prevention/correction of hyperventilation, and avoidance/treatment of hypotension) was not associated with significant improvement in overall survival to hospital discharge across the entire, combined moderate to critical injury spectrum. However, adjusted survival doubled among patients with severe TBI and tripled in the severe, intubated cohort [10]. These findings support the prehospital guidelines that recommend aggressive prevention and proactive treatment of hypotension in severe TBI.

## Monitoring and Treatment in the Intensive Care Unit (ICU)

### ICP

Current treatment of severe TBI in intensive care mainly aims to prevent secondary damage in cerebral hypoperfusion and tissue hypoxia. Monitoring and treatment guidelines are still focused on intracranial pressure (ICP) control as the main manageable driver of altered cerebral flow and ischemia. Besides this “static” intracranial volume control, there is increased attention to “dynamic” abnormalities in cerebral volume, perfusion, oxygenation, and metabolism that are only partially or even unrelated to elevated ICP [11].

Several continuous neuromonitoring techniques are available after admission to intensive care [12], but multimodal clinical application and experience are still limited. The most commonly applied modality in clinical practice is ICP, together with CPP. CPP is defined as MAP minus ICP. Originally, ICP monitoring was advised to detect the progression of space-occupying cerebral lesions (SOL) together with brain imaging. Nowadays, these numbers and trends are, in addition, used to detect more subtle and dynamic derangements of intracranial physiology and reserves. With the support of these modalities and dedicated software, changes in intracranial volume, compliance, and cerebral perfusion can be estimated over time [13–15].

### CPP

The guidelines established by the Brain Trauma Foundation recommend maintaining ICP below 22 mm Hg and CPP within the range of 60–70 mm Hg for patients with TBI. To determine the “optimal” CPP target tailored to individual patients, the guidelines advocate considering cerebral autoregulation status, as indicated by Carney et al. [2]. This recommendation is based on the premise that brain tissue exhibiting swelling and with intact cerebral autoregulation may potentially benefit from elevated brain perfusion targets, such as a CPP target of > 70 mm Hg, as delineated in the management protocols in the consensus algorithm proposed by Chesnut et al. [16]. However, such an approach also entails inherent risks of exacerbating brain edema and hemorrhage. Therefore, any inclination to target a CPP exceeding 70 mm Hg should be accompanied by compelling justifications substantiated by meticulous monitoring and follow-up. The consensus algorithm among experts advises implementing an intermittent practical "MAP challenge" to evaluate autoregulation status at the bedside [16]. Nevertheless, the practicality, reliability, and representativeness of this bedside intervention in informing clinical decisions remain uncertain, as recently highlighted by Dietvorst et al. [17]. Although personalized management remains the ultimate objective, its feasibility and effectiveness may vary across different ICUs.

### Cerebral Autoregulation

The effects of intracranial hypertension on cerebral perfusion are explained by simple physiological mechanisms that resemble the law of electric circuits (the law of Ohm). In hydraulic analogy, cerebral blood flow is directly proportional to the driving pressure (i.e., CPP) and inversely proportional to the total (active) resistance of the vascular system influenced or regulated by cerebral autoregulation [18].

First, there is a direct influence of elevated ICP on the CPP as the main driving force. The influence of venous pressure is generally omitted, but this is probably not correct with clinical circumstances like positive pressure ventilation or (traumatic) venous sinus thrombosis [19, 20]. CPP is the driving force behind cerebral perfusion, but the appropriate level to prevent ischemia varies between patients and probably within a patient over time. There is also no international consensus where MAP should be zeroed in patients with TBI with ICP monitoring: at the right atrial (heart) level or the foramen of Monro (brain) level. In the latter case, a CPP is obtained, which is corrected for the hydrostatic pressure drop that occurs because the head is at least 30° upwards to minimize cerebral venous congestion [21, 22]. Aligning ICP and MAP at comparable levels is logical, though it could potentially lead to confusion among clinical teams regarding perfusion targets for other organs. Several observational studies in severe TBI have shown that long-standing elevation of ICP and consecutive decreases in CPPs are associated with increased mortality [23–27]. Current treatment guidelines in intensive care are, therefore, fully focused on these population-based physiological values [2, 28], although updated consensus protocols also advise incorporating focal brain oxygenation for individual treatments [16].

In the study by Mikkonen et al. [29], the authors emphasize the significant impact of ABP transducer placement on the measurement of MAP, particularly concerning the calculation of CPP. They highlight that conventional placement of the ABP transducer at the level of the right atrium can lead to discrepancies in MAP readings when the patient's head is elevated, resulting in either overestimation or underestimation of CPP. Conversely, physiologically appropriate placement of the transducer at the level of the foramen of Monro provides a more accurate representation of CPP, but it may underestimate systemic pressures, potentially leading to the excessive and unwarranted use of vasopressors and fluids. This discrepancy in transducer placement affects the interpretation of CPP values and subsequently influences clinical decisions regarding the management of perfusion to various other organs. On the other hand, Thomas et al. [30] emphasize the importance of standardized transducer positioning for accurate CPP measurement, particularly in the management of TBI. They recommend positioning the ABP transducer at the level of the middle cranial fossa, approximated by the tragus of the ear, to ensure consistency in CPP calculations. This recommendation contrasts with the conventional practice of positioning the transducer at heart level (phlebostatic axis), which may introduce errors due to the variability in the relationship between MAP at the heart and MAP at the brain. The councils advocate for explicit reporting of transducer positioning in research articles and endorse consistent transducer repositioning following changes in body elevation or position to maintain accurate CPP measurements. Both studies underscore the critical role of ABP pressure transducer placement in CPP measurement and highlight the need for standardized practices to improve the reliability and interpretation of CPP values in clinical settings. In the manuscript, we have also briefly addressed the variances in MAP measurement due to transducer placement (zeroing). Placing the ABP transducer at the Monro level allows for automated height correction (if the head is in an upright position). This method offers several advantages: It provides a more accurate depiction of perfusion pressure at the level of brain vasculature; Zeroing ICP at the brain level aligns both ABP and ICP at a similar level for calculating CPP. However, there are notable disadvantages: Introducing “organ-specific zeroing” levels may cause confusion among attending physicians and nurses; Monro-corrected ABP might require higher vasopressor doses, potentially leading to adverse effects in other organs like the lungs, kidneys, and heart.

The study conducted by Lele et al. [31] meticulously investigates the potential disparities arising from utilizing noninvasive versus invasive ABP measurement techniques, particularly accentuating the critical impact of zeroing level on resultant ABP data within neurocritical care contexts. Through their analysis, they notably highlight substantial variations in ABP readings contingent upon the positioning of the transducer or arm. When measured by invasive ABP, the MAP, systolic blood pressure (SBP), and CPP calculated at the level of the external auditory meatus are significantly lower than at the level of the phlebostatic axis (transducer or arm position).

This divergence underscores the significance of meticulous transducer placement in ensuring accurate hemodynamic assessments, particularly concerning cerebral perfusion. Such discrepancies are not merely academic but carry significant clinical implications, as they can directly influence treatment decisions and patient outcomes.

**Fig. 1:**
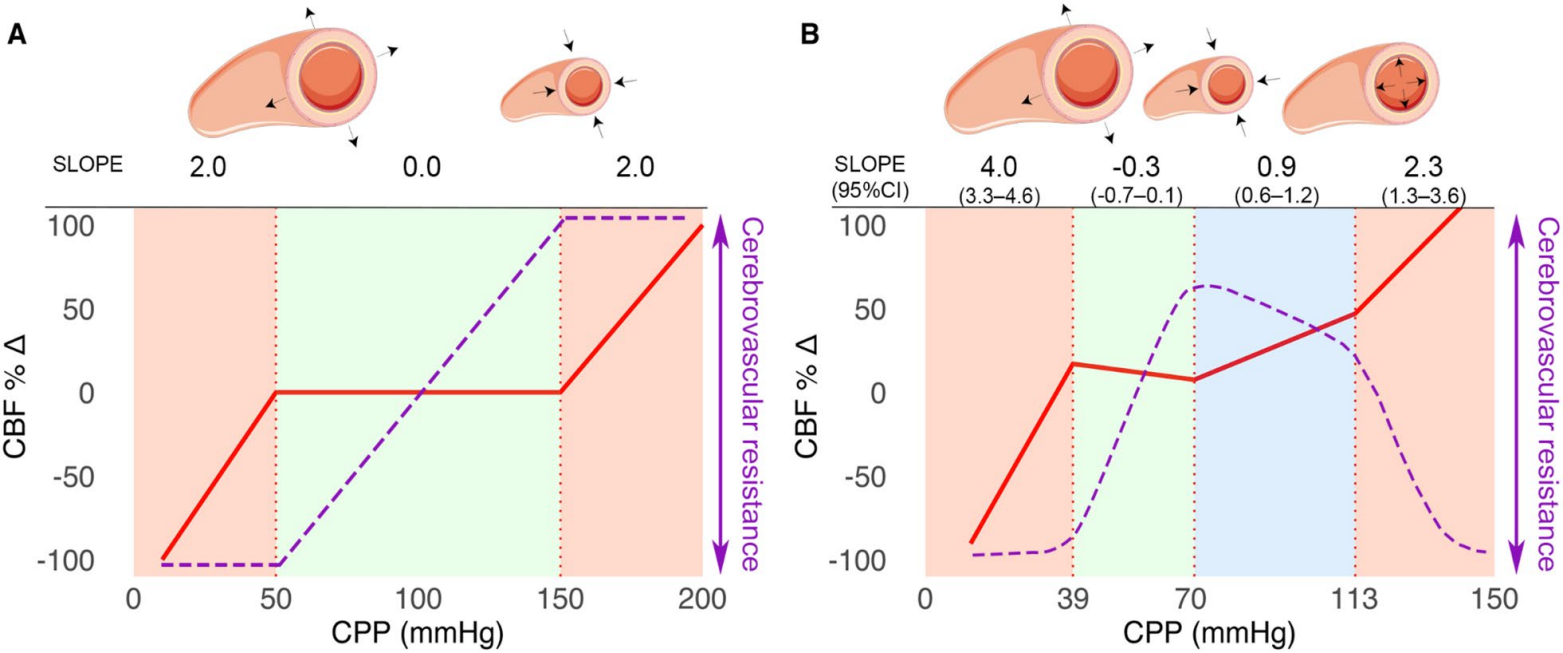
Schematic Representation of Cerebral Autoregulation with Cerebral Perfusion Pressure (CPP)–Cerebral Blood Flow (CBF) curve. **A** Traditional representation of cerebrovascular autoregulation (CA) depicts a triphasic curve denoted by a broad plateau of stable cerebral blood flow (CBF) (red curve) within a cerebral perfusion pressure (CPP) range of 50 to 150 mm Hg (green area). Regions in red signify absent CA, indicating a passive CPP-CBF relationship. Cerebrovascular resistance is indicated by the purple dashed line, and the physiological basis involves maximal vasodilation at the lower limit of autoregulation (LLA) and maximal vasoconstriction at the upper limit of autoregulation (ULA). **B** Recent animal model demonstrating a new quadriphasic curve featuring a relatively narrow plateau between LLA and ULA1 (green area). Progressive CA failure occurs between ULA1 and ULA2, starting at the smallest arterioles and extending toward larger arterioles (blue area), leading to complete pressure passive flow in all arterioles (right-sided red area). During maximal vasoconstriction (blue area), the CPP-CBF relationship is sloped but attenuated. Figure used with the permission of Klein et al. [33] [https://doi.org/10.1161/JAHA.121.022943] (Color figure online)

Second, for reasons not yet fully elucidated, cerebral autoregulation—which is essential to maintain adequate cerebral blood flow supply—is often disturbed both locally and diffusely in severe TBI. Cerebral autoregulation is a complex process in which it is believed that by vasoconstriction or vasodilation of the cerebral resistance vessels, cerebral perfusion is regulated over a wide range of MAP/CPP values. Other organs, such as the kidneys and lungs, also have an autoregulatory system, but the system is most intricate and elaborate within the high metabolic brain as tolerance of hypoperfusion is minimal, leading almost directly to synaptic dysfunction or neuronal cell death [32]. This unique protective mechanism is often presented as a broad plateau of constant cerebral perfusion with a “passively” decreasing cerebral perfusion with hypotension (denoted as the lower limit of autoregulation, LLA) and a “passively” ascending cerebral perfusion in hypertension on the other side (upper limit of autoregulation, ULA) (Fig. 1).

Following the above-described concept, with too low CPP levels (maybe < 50 mm Hg), there is a risk of hypoxia and ischemia, while excessively high CPP (maybe > 90–110 mm Hg) might lead to brain edema, intracranial hypertension, and possibly breakthrough hemorrhages and epilepsy. In animal studies, the relationship between CPP and cerebral perfusion has been reproduced, especially for the LLA [33]. This static and “safe” range of CPP values has been criticized, and suggestions have been made to include individual factors that might not only differ between patients (e.g., history of hypertension, PaCO_2_ levels) but also within patients over time (e.g., autoregulation reserve, intensity, and duration of physiological insults) [34]. As highlighted in Table 1, a growing body of clinical studies over the past 10 years emphasizes the upcoming role of cerebral autoregulation in guiding individual ABP/CPP management.

**Table 1:** Key clinical studies on cerebral autoregulation-guided ABP/CPP management (retrospective and prospective design) over the past 10 years in TBI patients

Author, year	Number of patients	Methodology/study design	Neuromonitoring variable/CA-Index	Key findings/outcomes
Agrawal et al., 2023 [47]	135	Prospective observational	ICP, CPP/PRx	Study protocol to study cerebral autoregulation using PRx in pediatric traumatic brain injury
Depreitere et al., 2014 [94]	201	Retrospective observational	ICP, CPP/CPPopt, long-PRx	Low-frequency data using a new method (minute-by-minute data) facilitates CPPopt calculation in ICUs
Donnelly et al., 2017 [40]	729	Retrospective observational	ICP, CPP/CPPopt, PRx	Personalized autoregulation-guided CPP management may replace fixed thresholds in severe TBI
Donnelly et al., 2018 [95]	231	Retrospective observational	ICP, CPP/CPPopt, PRx	In severe TBI patients, mortality is associated with prolonged periods of patients’ CPP below individualized CPPopt
Donnelly et al., 2019 [96]	1146	Retrospective observational	ICP, CPP/PRx	The implementation of an ICP/CPP protocol influenced ICP and CPP value over the years, but PRx has shown no significant improvements
Guilfoyle et al., 2021 [72]	619	Prospective observational	CPP, ICP, PbtO2/PRx	Elevated lactate/pyruvate ratio (LPR) within the first 3–7 days post-TBI predicts poor outcomes. Significant associations were observed between LPR and cerebral glucose, CPP, and PRx
Howells et al., 2018 [97]	104	Retrospective observational	ICP, CPP/CPPopt, PRx	Differences in CPPopt calculation was found between three European centers due to differences in demographics (age) and chosen CPP targets
Kramer et al., 2019 [98]	71	Retrospective observational	ICP, CPP/CPPopt, PRx	A clear association between ΔCPP (calculated as CPP-CPPopt) and clinical outcome was not found. The percentage of time spent below CPPopt increased significantly over time among patients with poor outcomes. This effect was magnified in patients with PRx > 0.2
Tas et al., 2021 [45]	60	Prospective interventional	ICP, CPP/CPPopt, PRx	Individualized and dynamic cerebral autoregulation-guided CPP targeting is feasible (47% of time patients’ CPP was concordant with CPPopt target) and safe in patients with TBI. Randomized control study
Petkus et al., 2020 [99]	81	Prospective observational	ICP, CPP/CPPopt, PRx	Averaged ΔCPP > 5.0 mm Hg, averaged PRx > 0.36, and LCAI > 100 min were significantly associated with mortality for the younger TBI patients. The critical values of averaged PRx > 0.26 and LCAI > 61 min were significantly associated with mortality for the elderly group
Wettervik et al., 2019 [41]	362	Retrospective observational	ICP, CPP/CPPopt, PRx	Higher PRx values and increasing monitoring time with CPP > CPPopt, but not the traditional fixed CPP thresholds, were strong predictors for worse clinical outcomes
Zeiler et al., 2019 [42]	204	Prospective observational	ICP, CPP/CPPopt, PAx, PRx, RAC	Unlike PAx, CPPopt parameters derived from PRx and RAC effectively predict 6 to 12-month outcomes in moderate to severe adult TBI

The search criteria for PubMed included the terms "traumatic brain injury," "cerebral autoregulation," and "(perfusion targets OR blood pressure targets OR cerebral perfusion pressure targets)", with additional filters for a 10-year timeframe, adult study participants (19 + years), English language, and human studies. The search yielded 29 relevant results

*ABP* Arterial Blood Pressure; *AMP* amplitude of ICP; *CPP* Cerebral Perfusion Pressure; *CPPopt* “Optimal” CPP; *ICP* Intracranial Pressure; *LCAI* longest period of cerebral autoregulation impairment; *MAPopt* “Optimal” Mean Arterial Pressure; *PAx* Pulse Amplitude Index; *PRx* pressure reactivity index; *RAC* correlation (R) between AMP (A) and CPP; *TBI* traumatic brain injury; *CMD* cerebral microdialysis

**Fig. 2:**
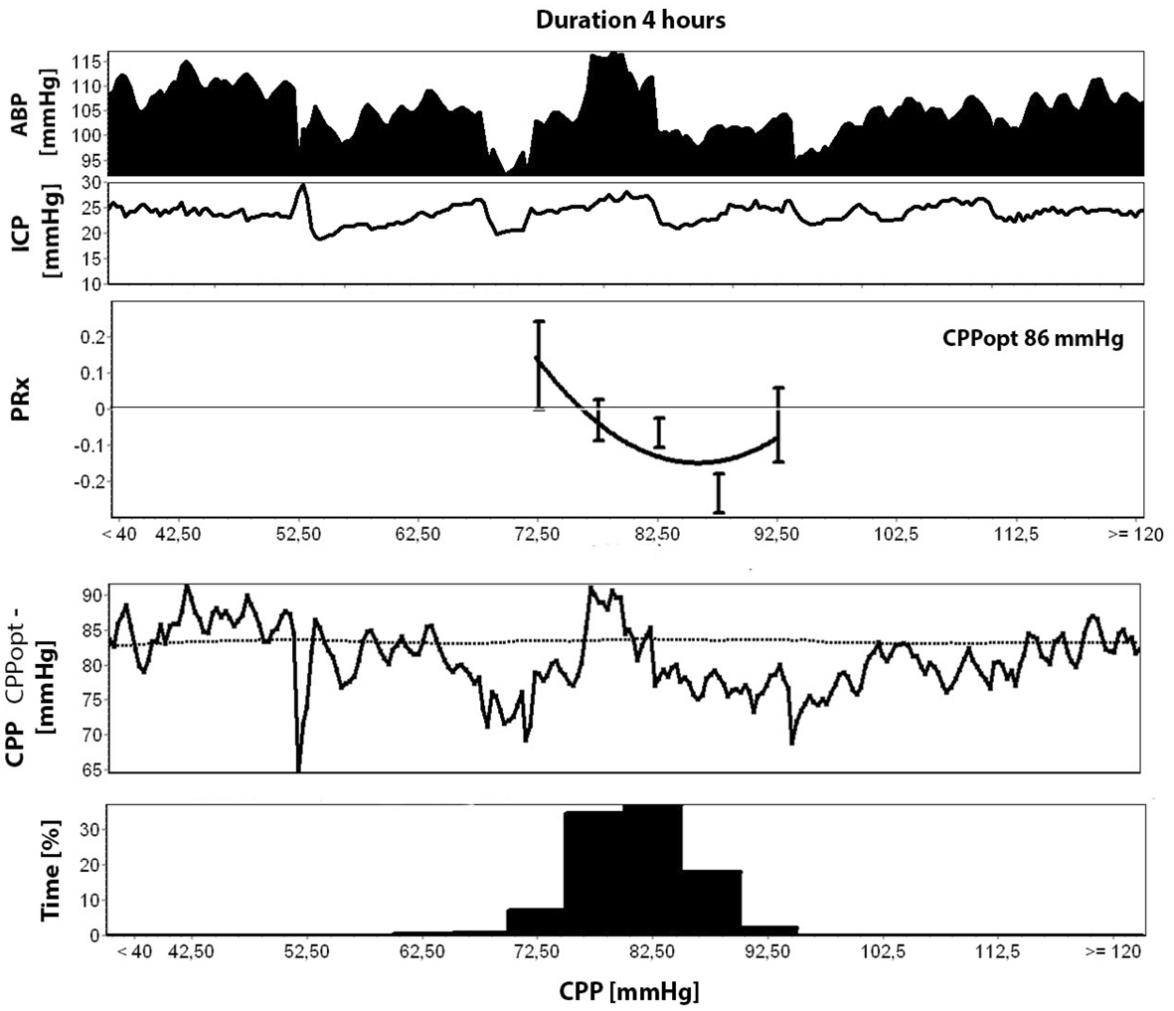
An example of intracranial pressure (ICP) and arterial blood pressure (ABP) trend lines over a 4-h monitoring period in a severe traumatic brain injury patient. The boxplot features the pressure reactivity index (PRx) plotted against 5 mm Hg intervals of cerebral perfusion pressure (CPP), including a fitted curve and automated “optimal” value for this particular patient (CPPopt of 86 mmHg). In the lower segment of the figure, the histogram represents the percentage of time (%) during which patients' CPP resided within predefined 5 mm Hg CPP intervals

In the recent SIBICC TBI consensus guidelines, a tiered treatment scheme is proposed, including a so-called MAP challenge [16]. The experts suggest evaluating carefully whether a period of higher MAP values (10–mm Hg increase, observation maximally 20 min) results in cerebral vasoconstriction, indicating intact cerebral autoregulation. Vasoconstriction will lead to a lower cerebral blood volume state, which results in lower ICP values. After a positive MAP challenge at the bedside, maintaining higher MAP/CPP targets for a longer period is advised, but re-evaluation is necessary [17]. Alternatively, these intermittent measurements might be replaced by continuous measurements of cerebral autoregulation, for example, by looking at the relationship between slow waves in MAP and ICP (the so-called pressure reactivity index, PRx). This concept can inform the clinician at the bedside at regular intervals at which CPP value autoregulation functions best for an individual patient (so-called “optimal” CPP or CPPopt target, Fig. 2). At present, the outcome benefit of these dynamic targets still needs to be proven [35], and comparisons with results from the MAP challenge are lacking.

## Guiding Cerebral Physiology and Perfusion Pressure Through Multimodal Brain Monitoring Modalities

Besides ICP, other more advanced options, including brain tissue oxygenation (PbtO2), near-infrared spectroscopy (NIRS), and transcranial Doppler (TCD), have been used as well to guide cerebral physiology in patients with severe TBI [36]. The SIBICC consensus working group recommends that PbtO2 could be an important additional variable monitored after ICP in moderate to severe patients [16]. However, PbtO2 results still hold room for interpretation and clinical benefit awaiting positive results from prospective studies [37]. Several homeostatic processes contribute to the adequate delivery of oxygen- and nutrient-rich arterial blood to the brain to match metabolic demand, as discussed in a recent review by [18]. These processes operate at distinct frequencies to precisely regulate cerebral perfusion. At the same time, cerebral microcirculatory flow is influenced by metabolically driven neurovascular coupling, oxygen and carbon dioxide levels, and glucose concentrations. Research findings support the existence of a "neurovascular complex" comprising functionally diverse modules distributed across segments. These modules work together to execute synchronized cerebrovascular responses to both "central" and "peripheral" signals, ensuring the maintenance of brain homeostasis [18]. It is important to recognize that, at present, in most clinical scenarios, a totally impaired vascular response is assumed, and hemodynamic control is limited to setting ABP or CPP targets [2].

Different monitoring modalities have been used to set ABP or CPP targets in patients with TBI more carefully, either using invasive or noninvasive options. Invasive and non-invasive monitoring guiding options are ICP, PbtO2, TCD, NIRS, and cerebral microdialysis (CMD). To our best knowledge, superficial and deep EEG, electrocorticography, brain temperature, regional cerebral blood flow, and jugular bulb venous oximetry have not been used to guide ABP or cerebral perfusion in patients with TBI.

### ICP

Pressure autoregulation of cerebral blood flow is an important homeostatic process that maintains a steady blood supply to the metabolically demanding brain. When CPP is below or above the limits of autoregulation, cerebral blood flow becomes pressure-passive or directly dependent on the driving pressure. CPPopt might be a physiologically plausible target to limit the risk of hypoperfusion and hyperperfusion. Retrospective data have shown an improved outcome in patients whose CPP values are concordant with the calculated CPPopt value [38–42]. Recent research results from the feasibility and safety COGiTATE (CPPopt-Guided Therapy Assessment of Therapy Effectiveness) randomized controlled trial pave the way for more individualized treatment options. Updated CPP recommendations (CPPopt) were presented to the clinical team four hourly in the intervention arm and compared to fixed CPP threshold management (60–70 mm Hg range). Using a specially designed software equipped with a multiwindow, weighted algorithm, CPPopt values were automatically computed to calculate the CPP for the most effective functioning of cerebral autoregulation (using ICP derived PRx) [43, 44]. In this approach, patients with TBI only required ICP/CPP monitoring, making this therapy immediately available in most middle- to high-income countries. Although the authors showed the feasibility and safety of PRx-based autoregulation-guided therapy, clinical benefit needs to be proven in larger outcome studies [45]. This potential treatment option is now also under investigation in pediatric TBI and other pathologies [46, 47].

### Transcranial Doppler Ultrasonography

TCD ultrasonography stands out as the most commonly employed noninvasive method [12]. It serves dual purposes, facilitating the early identification of cerebral hemodynamic issues such as increased cerebral edema, SOL expansion, and delayed cerebral ischemia/vasospasm while also aiding in decision-making for selecting patients for invasive monitoring [48]. TCD can be used to noninvasively estimate cerebral blood flow velocity (CBFV), intracranial hypertension, intracranial compliance, and cerebral autoregulation status at regular time intervals. CBFV real-time data provide insights into how changes in MAP affect cerebral perfusion, but assumptions regarding constant insonating vessel diameter must be taken into account. Colleagues from Argentina released a protocol for Ultrasound-Guided Cardio-cerebral Resuscitation (UGCeR) in patients with severe TBI.

In response to a pulsatility index > 1.4 and diastolic CBFV < 20 cm/s, indicative of a low cerebral perfusion state, their recommendation involves incrementally elevating MAP in 10 mm Hg increments (up to 120 mm Hg) and regularly reassessing TCD values. The protocol has not been evaluated yet with clinical outcome endpoints. Frequent measurements are, however, time-consuming, user-dependent, and not possible in every patient due to the absent bone window [49]. Short TCD recordings have been used to calculate autoregulation indices and “optimal” MAP targets in cardiac surgery patients undergoing cardiopulmonary bypasses (CPB) to avoid harmful cerebral perfusion fluctuations. Optimizing patients’ MAP to be greater than the individual patient’s LLA during the surgery significantly reduced the incidence of postoperative delirium [50]. So far, there is only limited experience with longer applications of robotic TCD recordings and the feasibility of determining TCD-based CPPopt targets in patients with TBI [51].

### NIRS

**Fig. 3:**
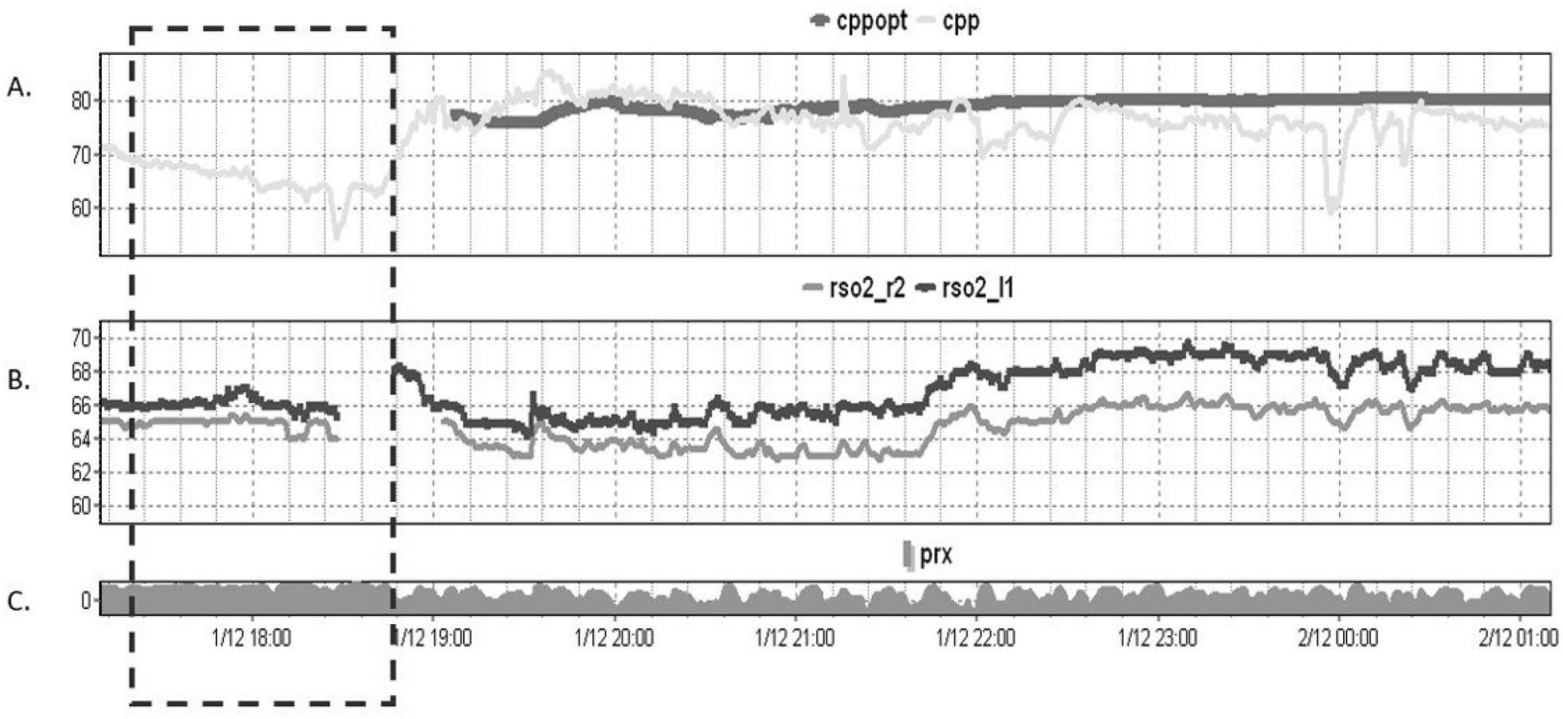
An example of a severe TBI patient undergoing invasive monitoring of ICP, CPP, and non-invasive bilateral frontal NIRS. **A** The trend of CPP (gray line) and CPPopt (black line) was observed over an 8-h recording period. No CPPopt calculation was possible during the period marked by a dashed box due to severe impairment in cerebrovascular reactivity (PRx close to + 1, as shown in C). However, cerebrovascular reactivity gradually recovered, and the trend of CPPopt indicated the necessity of maintaining higher CPP levels around 80 mmHg. **B** Both trends in NIRS regional oxygen saturation (rSO_2_) numbers increased over time, particularly with higher CPP levels, suggesting slow improved frontal oxygenation. *TBI* traumatic brain injury; *ICP* intracranial pressure; *CPP* cerebral perfusion pressure; *NIRS* near-infrared spectroscopy; *CPPopt* “optimal” cerebral perfusion pressure; *PRx* cerebrovascular pressure reactivity; *rSO*_*2*_ regional oxygen saturation; *r2/l1* right NIRS optode/left NIRS optode

NIRS is a noninvasive monitoring technique that uses light in the near-infrared spectrum to measure changes in the concentration of oxygenated and deoxygenated hemoglobin in the tissue beneath the optodes. Measuring changes in regional hemoglobin concentrations (rSO_2_, %), as reflections in the mismatch between cerebral oxygen supply and metabolic demand, seems attractive (Fig. 3). The rSO_2_ calculation essentially provides the proportion of oxygenated hemoglobin relative to the total hemoglobin in the tissue being monitored. Significant decreases or negative trends were associated with secondary brain damage and poor outcomes after acute brain injury [52]. The results from the 2016 NORMOSAT study confirmed that the use of a physiologic NIRS-based algorithm was a feasible approach to successfully manage intraoperative reductions in rSO_2_ in high-risk surgery patients. Early intervention with the algorithm—triggered by a 10% decrease in rSO_2_ value relative to baseline—resulted in an improved rate in reversal of oxygen desaturation, a positive effect on further progression of cerebral oxygen desaturation to a “clinically significant desaturation” of 20%, and a decrease in overall cerebral oxygen desaturation burden. In the initial stage of the NORMOSAT treatment algorithm, the MAP levels were assessed, and any hypotension was addressed by adjusting the MAP within 20% of the baseline value. NIRS can also be used to derive several indices related to changes in brain perfusion, hypoxia, intracranial volume, autoregulation, and metabolism. Assessment of CPPopt using the NIRS-based (volume) index total hemoglobin index (THx) was possible in only 50% of recordings and showed a significant agreement with the ICP-based CPPopt in patients with severe TBI [53]. NIRS may be of diagnostic value to “optimize” therapy oriented toward restoration and continuity of cerebral autoregulation, especially in patients for whom invasive ICP monitoring is not feasible [54]. Excursions of the MAP below the limit of NIRS-based autoregulation cerebral oximetry index (COx) and not absolute MAP values were independently associated with postoperative acute kidney injury (AKI) in pediatric cardiothoracic surgery patients on cardiopulmonary bypass (CPB). In pediatric CPB surgery patients, an association between an impaired pressure autoregulation burden and elevation in the serum brain damage biomarker (GFAP) was found [55–57]. However, it should be remembered that the NIRS method has numerous limitations, resulting mainly from unknown applied rSO_2_ calculation algorithms and its physical properties that are heavily influenced by extracerebral tissue, including intracranial/extracranial hematoma and pneumocephalus [58]. Therefore, the utilization of NIRS-guided perfusion treatments has been applied sparingly in patients with TBI.

### Brain Tissue Oxygenation

PbtO2 assesses oxygen levels within the brain's interstitial tissue, influenced by oxygen movement through the extracellular space. This movement is governed by a balance between oxygen supply at the capillaries and cellular consumption, driven by a concentration gradient. A PbtO2 threshold of < 20 mm Hg is considered a trigger for treatment and interventions, especially in patients with severe TBI [59]. Hirschi et al. [60] showed however that the time below treatment thresholds (‘hypoxia burden’) was more strongly associated with outcome than the mean PbtO_2_. A threshold of 19 mmHg most robustly distinguished patients by outcome, especially from days 3–5. However, additional benefit was suggested from maintaining values at least as high as 33 mm Hg [60]. A retrospective study by Kunapaisal et al. [61] investigated whether increasing MAP improved PbtO2 in patients with severe TBI with combined ICP, intermittent TCD, and PbtO_2_ monitoring. MAP augmentation resulted in four PbtO2 responses: normal and maintained (group 1: 58.5%), normal and deteriorated (group 2: 2.2%; average 45.2% PbtO_2_ decrease), low and improved (group 3: 12.8%; average 44% PbtO_2_ increase), and low and not improved (group 4: 25.8%). This probably explains that overall, no correlation between MAP or CPP and PbtiO_2_ was found [61].

**Fig. 4:**
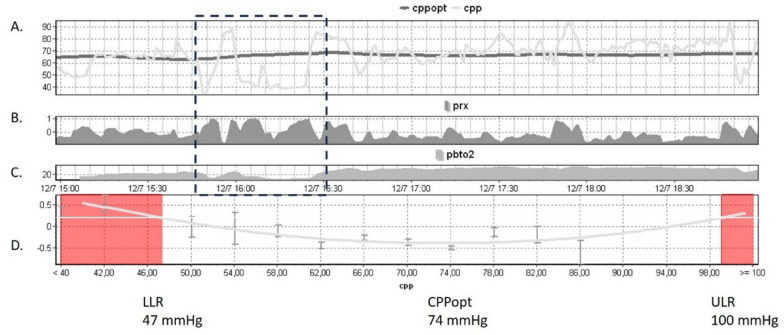
A severe TBI patient undergoing monitoring of ICP, CPP, and PbtO2. **A** Trends of CPP (gray line) and CPPopt (black line) over a 5-h period are depicted. **B** The trend of PRx shows values hovering around zero or negative, except during intervals of low CPP values (associated with decreased ABP, particularly highlighted in a 30-min period (dashed box), likely indicative of maximal vasodilation. Concurrently, CPP levels decrease to approximately 40 mm Hg. **C** During this “unstable” CPP period, PbtO2 values decline to below 10 mm Hg. **D** The CPPopt curve, utilizing PRx as an input variable, suggests an “optimal” CPP value of approximately 74 mm Hg for this patient. The LLR and ULR are automatically derived from this curve, with a PRx value of 0.2 serving as a threshold for impaired cerebrovascular pressure reactivity. *TBI* traumatic brain injury; *ABP* arterial blood pressure; *ICP* intracranial pressure; *CPP* cerebral perfusion pressure; *PbtO2* brain tissue oxygenation; *PRx* pressure reactivity index; *LLR* lower limit of vasoreactivity; *ULR* upper limit of vasoreactivity; *CPPopt* "optimal" cerebral perfusion pressure

A recent review summarized the current—mostly retrospective—evidence regarding the impact of PbtO2-guided therapy on the outcome of patients with TBI. When compared with ICP-guided treatment, the use of combined PbtO2/ICP-guided therapy was associated with a higher probability of favorable neurological outcomes and hospital survival [62]. It is worth noting that in the Brain Oxygen Optimization in Severe Traumatic Brain Injury II (BOOST-II) feasibility and safety trial, CPP augmentation above 70 mmHg was a treatment option for elevated ICP only in the combined PbtO2 and ICP monitoring group (a total of 41 instances of CPP augmentation recorded in the 57 patients) [63]. In that respect, this trial might be similar to the one that investigates CPP-oriented therapy (Fig. 4). In the recently released French OXY-TC randomized controlled trial, monitoring ICP and PbtO2 with absolute thresholds set at > 20 mm Hg for PbtO2 and < 20 mm Hg for ICP did not result in a decrease in the proportion of patients experiencing poor neurological outcomes at the 6-month mark. However, when conducting a post-hoc analysis on patients exhibiting ICP exceeding 20 mm Hg on ICU admission, a significant decrease in poor outcome scores was observed in the group treated with both ICP and PbtO2. This is somewhat unexpected, given that the recommended treatment for high ICP was comparable for both groups. It might point to the importance of correct patient selection who are likely to benefit from a multimonitoring guided treatment approach. From a practical site, instances of technical failures associated with the intracerebral catheter and intracerebral hematoma were more prevalent in the ICP and PbtO2 groups [64].

The different results of OXY-TC and BOOST-II concerning long-term outcomes are incompletely understood. Of note, the algorithms proposed for the treatment of low PbtO2 differed largely in the hierarchy of sequential items, which may be the key to further understanding. Within the context of the OXY-TC protocol, when a patient showed elevated ICP alongside decreased PbtO2, the protocol advised initially addressing ICP before turning attention to enhancing specific PbtO2 interventions. Currently, two randomized controlled trials investigating the impact on clinical outcomes of PbtO2-guided therapy in patients with TBI are ongoing: the BOOST-III and the Brain Oxygen Neuromonitoring in Australia and New Zealand Assessment Trial (BONANZA) [65, 66].

While awaiting further results of these trials, current recommendations and clinical algorithms for the management of TBI counsel that, with ICP therapy escalation, additional PbtO2 monitoring could be considered to get feedback on, for example, different CPP targets, “safe” PaCO2 lowering, and higher hemoglobin values [8].

PbtO2 produced a triphasic curve in patients with severe TBI when plotted against CPP like previous population-based plots of cerebral blood flow versus CPP (the so-called Lassen curve). The triphasic curve included a plateau region flanked by regions of relative ischemia (hypoxia) and hyperemia (hyperoxia). The plateau region shortened when autoregulation was disrupted compared to when it was intact [67]. In this large single-center retrospective TBI study, low PbtO2, under 25 mm Hg and particularly below 15 mm Hg, for longer durations and in combination with individual disturbances in global cerebral physiological variables (like CPP < 60 mm Hg or PRx > 0.3) were associated with poor outcome and may indicate detrimental cerebral hypoxia [67]. Prospective trials are required to determine whether personalized PbtO2-directed therapy is beneficial, identify the individual PbtO2 threshold at which interventions are justified, and how this may depend on the presence or absence of concurrent cerebral physiological disturbances like cerebral hypoperfusion or impaired autoregulation [68].

### Cerebral Microdialysis

Microdialysis is an invasive technique used in ICUs to continuously monitor and sample brain extracellular fluid, providing real-time data on cerebral metabolism [69]. This method allows for the bedside analysis of various metabolites, including glucose, lactate, pyruvate, glutamate, and glycerol. Research supports that the absolute levels, ratios, and temporal patterns of these metabolites can indicate cerebral metabolic dysfunction and highlight potential intervention targets. Among these parameters, the lactate/pyruvate ratio (LPR) is the most thoroughly investigated. It serves as a key marker of the cellular redox state and the balance between aerobic and anaerobic metabolism. Elevated LPR values, specifically those exceeding thresholds of 25 or 40, are associated with higher mortality rates and poorer outcomes following TBI [69]. Furthermore, low brain glucose levels, around 0.8–1 mM, are linked to adverse outcomes, as demonstrated in observational studies and trials comparing different glycemic control strategies. Conversely, excessively high brain glucose levels can also be detrimental to neuronal health [69, 70]. In a large single-center study, significant nonlinear associations were observed between reduced LPR and higher CPP levels (> 70 mm Hg), working autoregulation (PRx < 0.1), higher PbtO2 and brain glucose levels (> 18 mm Hg and > 1 mM, respectively). Preliminary studies have been directed at incorporating physiological interventions to treat deranged microdialysis parameters, but these are yet to be proven to be clinically beneficial [71]. Future prospective studies are required to determine the efficacy of strategies to improve cerebral metabolism and outcome [72].

## Strategic Navigation of Continuous Targets Through Guided Monitoring

Achieving MAP or CPP targets in individual patients with TBI often involves a gradual and methodical approach, with frequent reassessment. The suggested lower limit for CPP is often set at 50 mm Hg for TBI, grounded in safety considerations related to “historical” cerebral autoregulation thresholds from the Lassen review. Concerns also arise among clinicians with CPP levels surpassing 100 mm Hg, as many clinicians are worried about potential complications such as cerebral edema, worsening of hemorrhages, and adverse systemic effects of vasopressors and fluids (organ ischemia or pulmonary adult respiratory distress syndrome [ARDS]) [73, 74]. Therefore, the playing field for neuromonitoring-guided treatment seems to be somewhere between CPP levels of 50–100 mm Hg, although no formal “safety” recommendations have been published [43]. Interestingly, management of TBI using CPPopt-guided therapy was associated with better outcomes in a single-center observational study and seems to be safe regarding the development of secondary lung injury [75].

To achieve CPP targets, intravenous fluids are often administered together with vasopressors to increase MAP levels. Guidelines stress maintaining euvolemia, with a keen awareness of avoiding excessive resuscitation, which can contribute to increased ICP and systemic complications like coagulopathy and metabolic abnormalities. Vasopressors, intravenous drugs that induce peripheral (venous and arterial) vasoconstriction, are used in most patients with TBI to counteract vasodilation of sedatives and maintain CPP levels.

To understand the intricacies of modulation, clinicians must carefully consider variables such as vasopressors, fluids, inotropes, and the need for red blood cell transfusion. Recognizing that modulation of ABP is probably the key avenue, it becomes crucial to weigh the options, considering the nuances of each intervention and their potential impact on cerebral perfusion regulating mechanisms. An important observation in the COGITATE study was that although in the autoregulation-guided intervention arm, higher CPP targets were set, lower vasopressor dosages and fluid administration were needed. This underscores the potential advantage of a nuanced and individualized strategy in the pursuit of optimizing brain physiology and making use of the brain's own autoregulatory capacity.

## Disadvantages of Cerebral Perfusion-Focused Management

Optimizing cerebral perfusion through the adjustment of MAP, to some extent done aggressively, comes with potential benefits but is not without its drawbacks. First, there might be a negative direct impact on the brain parenchyma or physiology itself. In the CPP-guided approach, most patients end up with (periodically) high MAP levels (> 70 mm Hg). As an alternative, the so-called Lund concept has focused on the management of brain edema and ICP, along with the improvement of cerebral perfusion and oxygenation. The Lund concept was introduced in 1990 to 1991 at the University Hospital of Lund, Sweden. The initiation of the approach was prompted by the elevated mortality rate among patients with TBI at that time and the limited physiological and clinical support provided by the standard therapies in use [76]. The Lund therapy considers the consequences of a disrupted BBB for the development of brain edema, impaired autoregulation, and the specific consequences of a rigid dura/cranium for general cerebral hemodynamics. The approach has been criticized because these mechanisms are assumed to be severely impaired or exhausted without attempts to measure it (serially) [77]. Lund therapy is achieved by normal blood oxygenation, by maintaining normovolemia with normal hematocrit and plasma protein concentrations, and by antagonizing vasoconstriction through reduction of catecholamine concentration in plasma and sympathetic discharge (minimizing stress and by refraining from vasoconstrictors and active cooling).

While the Lund concept holds promise for enhancing patient outcomes in critical care settings, it's essential to consider both its advantages and disadvantages. Grounded in a physiological theory, this approach offers a pathway to improving patient care by optimizing oxygen delivery to vital organs and tailoring treatment strategies to individual physiological parameters. Additionally, its potential to reduce or eliminate the need for vasopressors presents a compelling advantage, mitigating the associated risks of systemic adverse side effects.

However, several drawbacks have tempered this approach over time. Firstly, the Lund concept’s applicability beyond Scandinavia is limited, with its adoption remaining uncommon elsewhere. Moreover, its absence from international guidelines raises concerns about standardization and widespread acceptance. Furthermore, despite theoretical promise, large randomized controlled trials have yet to demonstrate clear clinical benefits, casting doubt on its efficacy in improving patient outcomes. Moreover, although there have been suggestions regarding impaired autoregulation and dysfunctional BBB, there is currently no inclusion of conclusive bedside measurements validating these concerns in the treatment algorithm. In summary, although the Lund concept may offer promise for certain patients with severe TBI experiencing specific physiological disturbances, such as dysfunctional BBB, its limitations and uncertainties necessitate that it be applied only in research settings.

Following the discussion of the Lund concept, it is crucial to consider the implications of well-known clinical trials such as those by Chestnut et al. [78] and Nattino et al. [79], which revealed no superiority in outcomes for patients with TBI with (early) continuous ICP-guided treatment protocol. The observational cohort in Nattino et al. [79] highlighted that early ICP monitoring was associated with poorer recovery and increased medical interventions; however, this analysis was likely confounded by “indication” bias (related to receiving early ICP monitoring). Conversely, excessive treatments driven by ICP monitoring, such as the administration of high doses of vasopressors to elevate CPP, may lead to unsatisfactory results. Therefore, it seems essential to recognize that an autoregulation-based management of brain perfusion targets is probably only applicable and beneficial within a specific pathophysiological model of secondary brain injury (e.g., low intracranial compliance, presence of autoregulation plateau).

Second, changing ABP might cause or aggravate extracranial injuries in other (affected) organs. Extracranial complications are common and influence the outcome of TBI. Significant improvements in outcomes in a sizable proportion of patients could potentially be accomplished by improving the ability to prevent or reverse nonneurological complications.

Prioritizing perfusion pressures for one organ may come at the expense of others, potentially leading to complications like ARDS, pneumonia, or AKI. Prompt recognition and treatment of systemic complications are therefore fundamental to the care of this patient cohort. So far, the role of extracranial pathology has often been underestimated in outcome assessment because most clinicians focus mainly on intracranial lesions and injury rather than consider the systemic effects of TBI [80]. Further studies are warranted to precisely understand and manage the multisystem response of the body after TBI and how individual treatment levels, such as CPP, should be adjusted.

## Role of Cardiac Output in the Management of Cerebral Blood Flow

The management of CBF encompasses a multifaceted approach integrating various physiological parameters, among which evaluating cardiac output (CO) and systemic vascular resistance plays a crucial role. The brain, with its dense vascular supply and high metabolic demand, relies on intricate mechanisms such as cerebral autoregulation to maintain adequate CBF [32]. Autoregulation dynamically adjusts cerebrovascular resistance in response to changes in ABP, ensuring stable CBF despite fluctuations in ABP levels [32]. The effect of CO on CBF has been reviewed [81]. A compilation of data from nonanesthetized individuals demonstrated that cerebral blood flow is correlated with CO via different mechanisms. Pressure autoregulation, however, likely remains functional despite shifting up or down of the CBF–ABP relationship. In contrast, data derived from animals and humans during CPB support have shown that ABP above a critical threshold (presumably the LLA) is the main determinant of adequate CBF, not systemic flow rate (equivalent of CO) [82, 83]. Notably, during CPB support, the CO can be manipulated without the administration of vasoactive drugs that may indirectly influence CBF. However, we must keep in mind that in multitrauma patients (including TBI), these regulatory and protective mechanisms may be under threat and all together exacerbate secondary brain injuries. This relates to possible important differences between patients with isolated brain injury and multiple traumatic injuries. Krishnamoorthy et al. [84] showed that early “multiple” organ dysfunction following moderate-severe TBI is common and independently impacts multiple domains (mortality, function, and disability) over the year following injury. Further research is necessary to understand underlying mechanisms, improve early recognition, and optimize management strategies [85]. The high prevalence of cardiac dysfunction (including QT elongation and Takotsubo cardiomyopathy) following neurologic injuries underscores the intricate interplay between the neuroendocrine system, neuroinflammation, and injury-related catecholamine release [86–88]. In an isolated TBI cohort, 22.3% had an abnormal echocardiogram, reduced left ventricular ejection fraction was documented in 12% (left ventricular ejection fraction, 43% ± 8%), and 17.5% of patients had a regional wall motion abnormality. Abnormal echocardiogram was independently associated with all-cause in-hospital mortality [89].

## Outlook and Future Perspectives

When contemplating the future perspective of managing patients with TBI, it is increasingly recognized that conducting research is crucial for developing a nuanced understanding of the parameters to focus on over time, particularly within specific patient populations [90]. To improve patient outcomes, embracing a “proactive” strategy is advocated to reduce the incidence of secondary brain injuries. The integrated utilization of neuromonitoring modalities alongside systemic variables, such as ABP/CPP, allows for a thorough and personalized approach over time. The prevailing trend in reviews and guidelines underscores the significance of tailored treatment options, endorsing personalized ABP/CPP targets that align with the individual patient’s specific status and requirements. The ongoing discussion revolves around the uncertainty of whether these targets should be derived from continuous “single” parameters that are widely available and well-understood (such as ICP, CPP, and associated autoregulation or reactivity indices) or from a “multimodal” brain monitoring approach encompassing (changes in) cerebral volume, cerebral oxygenation, cerebral metabolism, and cortical activity [91]. One prominent challenge is the lack of universally standardized protocols for interpreting and acting upon the physiological values obtained through (multimodal) neuromonitoring. This absence of structured guidelines can result in variability and uncertainty in clinical decision-making, potentially leading to suboptimal patient care. Moreover, interpreting neuromonitoring data can be complex and may require a high level of expertise, which is not always readily available in every clinical setting. For example, the Continuous Cerebral Autoregulation Monitoring in the Clinical Practice of Neurocritical Care and Anesthesia network was recently launched. The initiative calls for a strong effort by a group of clinical experts to transform the plausible benefits of autoregulation monitoring and guided treatments—already in use at some centers—into a more standardized and RCT-validated clinical reality [92]. It is crucial to emphasize that the current implementation of “autoregulation-guided ABP/CPP management” does not directly augment the autoregulatory function itself. Instead, it suggests that patients are moved toward a potentially “safer” zone within the autoregulatory spectrum, commonly referred to as the “middle of the cerebral autoregulation plateau or Lassen plateau.” The direct enhancement of autoregulatory function, particularly in terms of smooth muscle cell function, remains a subject largely confined to investigative efforts.

Looking ahead, further advancements in TBI research should prioritize enhanced data collection methodologies, utilization of advanced multimodal software platforms, incorporation of larger patient sample sizes, understanding the interaction between different signals, and the development of personalized interventions [61, 93]. This holistic approach aims to foster a dynamic and comprehensive understanding of effective strategies for managing ABP and CPP in patients with TBI, ultimately improving patient outcomes and enhancing clinical practice. The integration of bedside echocardiography into ICUs represents a significant advancement in medical technology, enabling real-time assessment of cardiac function. This hopefully helps in understanding the role of CO in the complex hemodynamic management of patients with TBI, which has so far focused largely on perfusion pressures.

## Narrative Review Criteria

PubMed database was searched for relevant articles published in English between January 2000 and January 2024 concerning the adult population. The following search terms were used: “blood pressure targets,” “hemodynamic management,” “cardiac output,” “cerebral perfusion pressure targets,” “invasive brain monitoring,” “non-invasive brain monitoring,” “traumatic brain injury,” “TBI,” “cerebral autoregulation,” “cerebrovascular autoregulation,” “regulation of cerebral blood flow,” and “outcome.” References were checked for additional material. Studies were considered when clinical implications for hemodynamic management in the emergency room, intensive care, and operating room were mentioned. A specific literature search was done for recent key articles concerning autoregulation-guided cerebral perfusion pressure CPP/ABP therapy in patients with severe TBI (see legend Table 1).
